# Critical role of DNA intercalation in enzyme-catalyzed nucleotide flipping

**DOI:** 10.1093/nar/gku919

**Published:** 2014-10-16

**Authors:** Jenna M. Hendershot, Patrick J. O'Brien

**Affiliations:** Department of Biological Chemistry, University of Michigan Medical School, Ann Arbor, MI 48109, USA

## Abstract

Nucleotide flipping is a common feature of DNA-modifying enzymes that allows access to target sites within duplex DNA. Structural studies have identified many intercalating amino acid side chains in a wide variety of enzymes, but the functional contribution of these intercalating residues is poorly understood. We used site-directed mutagenesis and transient kinetic approaches to dissect the energetic contribution of intercalation for human alkyladenine DNA glycosylase, an enzyme that initiates repair of alkylation damage. When AAG flips out a damaged nucleotide, the void in the duplex is filled by a conserved tyrosine (Y162). We find that tyrosine intercalation confers 140-fold stabilization of the extrahelical specific recognition complex, and that Y162 functions as a plug to slow the rate of unflipping by 6000-fold relative to the Y162A mutant. Surprisingly, mutation to the smaller alanine side chain increases the rate of nucleotide flipping by 50-fold relative to the wild-type enzyme. This provides evidence against the popular model that DNA intercalation accelerates nucleotide flipping. In the case of AAG, DNA intercalation contributes to the specific binding of a damaged nucleotide, but this enhanced specificity comes at the cost of reduced speed of nucleotide flipping.

## INTRODUCTION

Although DNA is a remarkably stable molecule, it is nevertheless subject to damage by a variety of reactive intracellular and environmental agents. DNA damage can alter gene expression, affect epigenetic profiles, and even cause cell death (1,2). To recognize and repair DNA, enzymes must gain access to damaged bases that are normally embedded in the duplex. This is aided by a process called nucleotide flipping that involves the complete 180° rotation of a nucleotide into the enzyme active site (3). It is proposed that both DNA bending and DNA intercalation are important for nucleotide flipping (3,4). Even though nucleotide flipping is prevalent among DNA repair enzymes, the mechanism is not well understood. In particular, the timing and the energetic contributions provided by DNA intercalation are not known.

**Figure 1.**
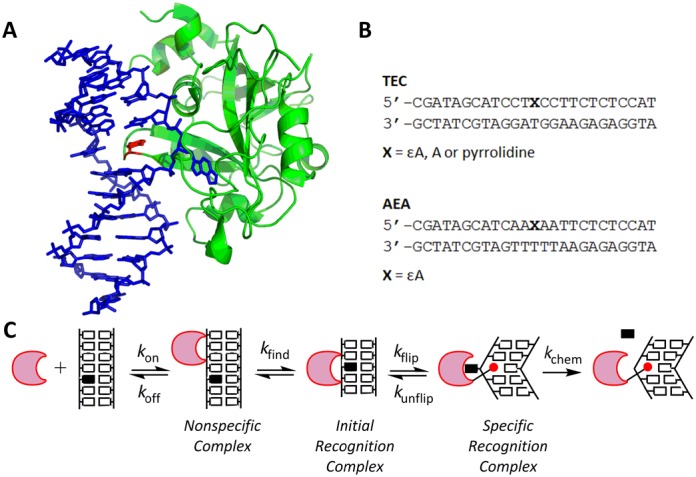
Nucleotide flipping by AAG. (**A**) Crystal structure of human alkyladenine DNA glycosylase (E125Q) bound to εA-containing DNA (14,19). Image was rendered with Pymol (http://www.pymol.org) using coordinates from the PDB (1EWN). The εA base is flipped into the active site, and the intercalating residue (Y162) is shown in red. (**B**) Sequences of the 25mer oligonucleotide substrates. (**C**) Minimal mechanism for the initiation of BER. AAG (crescent) binds to nonspecific DNA and rapidly searches for DNA damage. This searching process is described by the macroscopic rate constant *k*_find_. Once a lesion (solid rectangle) is encountered in an initial recognition complex, it can be flipped into the enzyme active site to form the specific recognition complex. In this specific complex, Y162 (red circle) intercalates into the DNA where it takes the place of the extrahelical lesion. AAG catalyzes hydrolysis of the *N-*glycosidic bond (*k*_chem_) from this complex.

Human alkyladenine DNA glycosylase (AAG), also known as methylpurine DNA glycosylase (MPG), is one of many enzymes that use nucleotide flipping to engage substrates. AAG initiates the base excision repair (BER) pathway by locating sites of damage and catalyzing the hydrolysis of the *N*-glycosidic bond to release the damaged base (5). This monomeric repair protein is responsible for removing a diverse set of alkylated and deaminated purine lesions (6–9). One of the lesions that is most efficiently excised by AAG is 1,*N*^6^-ethenoadenine (εA) that is formed by lipid oxidation or exposure to exogenous alkylating agents (9,10). The minimal kinetic mechanism for multistep recognition of εA damage has been reported, including the rate of nucleotide flipping, which was directly monitored by fluorescence to follow changes in the intrinsic fluorescence of εA (11,12). Given the excess of undamaged DNA, initial binding of AAG is not at the site of damage (Figure 1C). AAG uses nonspecific binding and facilitated diffusion (13) to search for sites of damage. These searching steps are expected to be very rapid and we define the macroscopic rate constant *k*_find_ to describe the productive searching process. Once an εA is found, AAG forms an initial recognition complex that is accompanied by changes in the stacking of the lesion base (11). From this intermediate, AAG catalyzes nucleotide flipping to form a more stable specific recognition complex that positions the substrate for *N*-glycosidic bond cleavage, which releases the damaged base (14). Multistep recognition provides multiple opportunities for discrimination between damaged and undamaged nucleotides and similar mechanisms have been proposed for independently evolved DNA repair glycosylases, including 8-oxoguanine DNA glycosylases and uracil DNA glycosylases (15–18).

Crystal structures of AAG bound to εA-DNA revealed that the DNA is bent in the specific recognition complex and a tyrosine (Y162) intercalates into the space left by the flipped-out damaged nucleotide [Figure 1A; (14,19)]. Similar intercalating interactions are observed in all nucleotide flipping enzymes, but the identity of the intercalating amino acid varies and the function(s) of the intercalating residue remains unknown (20,21). The two models for how DNA intercalation could contribute to nucleotide flipping have been described as ‘pushing’ and ‘plugging’ (22–24). In the pushing model, DNA intercalation destabilizes the DNA duplex to accelerate nucleotide flipping. In the plugging model, DNA intercalation occurs subsequent to nucleotide flipping and provides a barrier to the return of the nucleotide to the duplex, effectively slowing the rate of unflipping. These two mechanisms are not mutually exclusive, and both models can be addressed by kinetic studies of proteins in which the identity of the intercalating residue has been varied.

We investigated the functional contributions of the intercalating residue to each of the steps in the AAG catalytic mechanism by mutating the intercalating tyrosine residue to phenylalanine (Y162F) and alanine (Y162A). Remarkably, the Y162A mutation increases the rate of nucleotide flipping by 50-fold relative to wild-type (WT) AAG. This mutant also exhibits faster unflipping, resulting in a 140-fold reduction in the equilibrium constant for formation of the flipped-out complex. In contrast, the kinetic parameters for the Y162F and WT enzymes are very similar, suggesting that the hydroxyl of tyrosine is not necessary for function. These results establish that DNA intercalation contributes to the specific recognition of DNA damage by acting as a plug to stabilize the specific recognition complex. DNA intercalation stabilizes the initial recognition complex without providing a push, slowing the rate of nucleotide flipping. Nevertheless, tyrosine 162 plays a critical role in the discrimination between damaged and undamaged nucleotides, by increasing the amount of the extrahelical lesion recognition complex and enabling efficient repair of rare sites of damage.

## MATERIALS AND METHODS

The catalytic domain of human AAG was expressed in *Escherichia coli* and purified as previously described (25). The Y162F and Y162A mutants were constructed by site-directed mutagenesis, verified by sequencing both strands of the open reading frame, and purified using the same protocol as used for WT AAG. Oligonucleotides were purified as previously described (13). The standard condition for kinetic experiments was 50 mM NaMES (pH 6.5), 100 mM NaCl, 1 mM ethylenediaminetetraacetic acid (EDTA), 1 mM DTT and 25°C. Single-turnover glycosylase activity with fluorescein-labeled DNA was measured with a discontinuous assay that utilizes abasic site cleavage by NaOH followed by separation of fluorescein-labeled DNA on a denaturing polyacrylamide gel (11). Steady-state fluorescence emission spectra were collected with an excitation wavelength of 314 nm (6 nm band-pass) and emission wavelengths from 340 to 480 nm (6 nm band-pass). Stopped-flow experiments were performed on a Hi-Tech SF-61DSX2, controlled by Kinetic Studio (TgK Scientific). The fluorescence of εA was measured using an excitation wavelength of 313 nm and a WG360 long-pass emission filter (11,12). Macroscopic rate constant for dissociation of WT and mutant AAG from εA-containing DNA was measured by pulse-chase as previously described for WT AAG (11,12). Stopped-flow double-mixing experiments were performed to measure unflipping and dissociation of εA-DNA by Y162A as previously described (12). See Supplementary Material for detailed methods, kinetic equations, and global analysis of stopped-flow data using Berkeley Madonna (Berkeley Madonna Inc.).

## RESULTS

### Binding and excision of εA by AAG mutants

**Figure 2.**
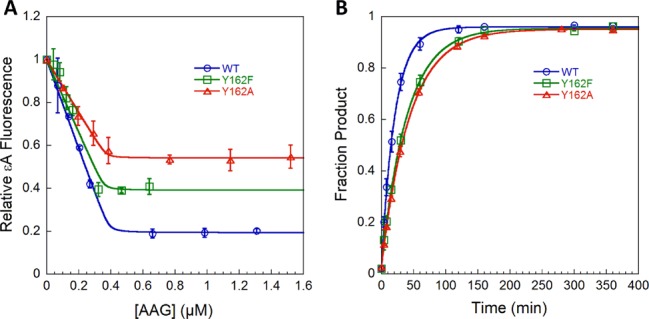
Binding and excision of εA by AAG. (**A**) Titration of 0.4 μM εA-DNA with WT and mutant AAG proteins. Steady-state fluorescence was monitored within 1 min, before excision can occur, and normalized by dividing by the fluorescence of free DNA. WT and mutants show tight binding to εA-DNA [Equation (S1)]. (**B**) Single-turnover excision of εA by WT and mutant AAG with 50 nM DNA and saturating (300 nM) enzyme was fit by a single exponential [Equation (S2)] to obtain the rate constants for *N*-glycosidic bond cleavage (Table 1). Reactions with 600 nM AAG gave identical rate constants, confirming that 300 nM was saturating (Supplementary Figure S1). Data points are the average ± SD from three independent experiments.

We used site-directed mutagenesis to create Y162F and Y162A variants, and characterized their ability to bind and excise εA from synthetic 25mer oligonucleotides containing a central εA·T mismatch. It was advantageous to use two different sequence contexts for these studies (AEA and TEC; Figure 1B), because the fluorescence of the εA is significantly higher in the AEA context than in the TEC context (11). As the εA fluorescence of the enzyme-bound intermediates is the same for the two contexts, the choice of DNA affects the magnitude and direction of the signals for binding and conformational changes. Previous work and the results herein suggest that AAG has similar kinetic parameters with these two oligonucleotides (11). AAG has a relatively slow rate of base excision; therefore, steady-state fluorescence titrations could be used to measure binding of active enzyme to εA-DNA (11). For this experiment, we used the AEA DNA, and the stable binding of the extrahelical lesion results in a 5-fold quenching of fluorescence for WT AAG (Figure 2A). Titrations of Y162A and Y162F AAG showed somewhat less quenching of εA-fluorescence (2–3-fold), but nonetheless indicate tight binding of εA-DNA. The similar 1:1 stoichiometry at the equivalence point for each of the proteins provides validation that the protein and DNA concentrations were accurately determined.

To evaluate if the mutant proteins retain catalytic activity, single-turnover experiments were performed with enzyme in excess over εA-DNA substrate (Figure 2B and Supplementary Figure S1). For WT AAG, the single-turnover rate constant reflects the *N*-glycosidic bond hydrolysis step (11). The rate constant for WT AAG (0.05 min^−1^) matches the previously reported value (11,12). The rate constant for excision of εA by Y162F (0.03 min^−1^) is similar to WT, consistent with reports that the Y162F mutation is well tolerated (26,27). We were surprised to find that the rate constant for excision of εA is only slightly smaller for Y162A AAG (0.02 min^−1^). The similar rate constants for base excision strongly suggest that these mutations do not disrupt the active site, but larger effects on recognition and flipping of the damaged base are likely to be masked by the high affinity binding of AAG to εA-DNA. Therefore, it is important to measure individual reaction steps to address the question of how DNA intercalation by Y162 contributes to recognition and flipping of damaged nucleotides.

### Stopped-flow fluorescence to monitor binding and flipping of εA-DNA

**Figure 3.**
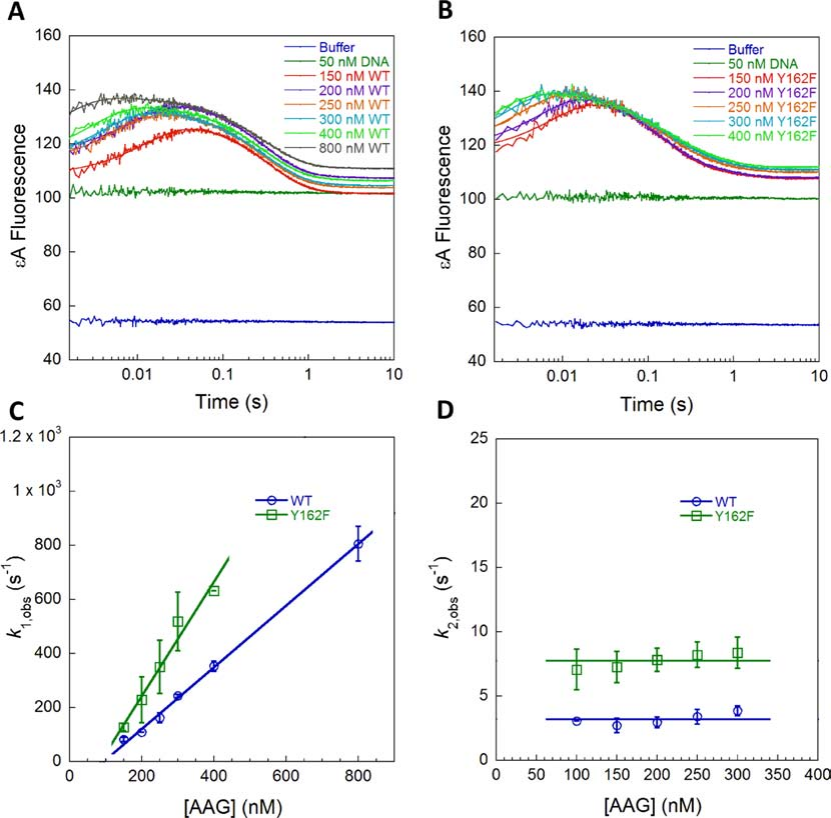
Stopped-flow fluorescence with excess protein to measure binding and nucleotide flipping by WT and Y162F AAG. Representative data from experiments in which 50 nM TEC DNA was mixed with WT (**A**) or Y162F (**B**). Traces are the average of three binding reactions and are fit by the sum of exponentials [Equation (S4)]. (**C**) The rate constant for the first phase of the binding reaction (*k*_1,obs_) is dependent on the concentration of AAG and a linear fit yields the bimolecular rate constant for binding (*k*_on_) with the values summarized in Table 1. It is notable that the intercepts are negative, similar to what has been observed in another case of extremely fast and tight binding. (**D**) The rate constants for the second phase of the binding reaction (*k*_2,obs_) are independent of the concentration of AAG and reflect the sum of the forward and reverse rate constants for nucleotide flipping. Rate constants in (C) and (D) are from three independent experiments (average ± SD).

We performed rapid mixing experiments of WT and mutant enzymes with εA-DNA to determine the rate constants for association and flipping. By improving the sensitivity of the stopped-flow, we were able to make measurements over a wider concentration range than was previously possible (11). When εA-DNA was mixed with excess WT AAG, we observed an initial increase in fluorescence that was followed by a decrease in fluorescence (Figure 3A). The first phase is linearly dependent on the enzyme concentration (Figure 3C) and this was previously assigned to binding and formation of the initial recognition complex (11). The second phase is independent of concentration and corresponds to nucleotide flipping to form the specific recognition complex. We sometimes observed a much slower third phase, with a small change in amplitude that may reflect an artifact such as photobleaching. This phase was not observed in all experiments and was not reproducible. When three phases were observed, we fit the traces by a triple exponential [Equation (S4)], because this gives the most consistent fits to the first two phases. Experiments with Y162F AAG yielded very similar results as the WT enzyme, with a transient increase in εA-fluorescence followed by a decrease in fluorescence (Figure 3B). The second order rate constant for the binding step is 2-fold greater for the Y162F mutant as compared to WT (Figure 3C). The intercepts for both WT and Y162F are negative, similar to what has been observed in another case of extremely fast and tight binding (28). The formation of the flipped-out specific recognition complex is approximately 2-fold faster for the Y162F mutant as compared to WT AAG (Figure 3D).

**Figure 4.**
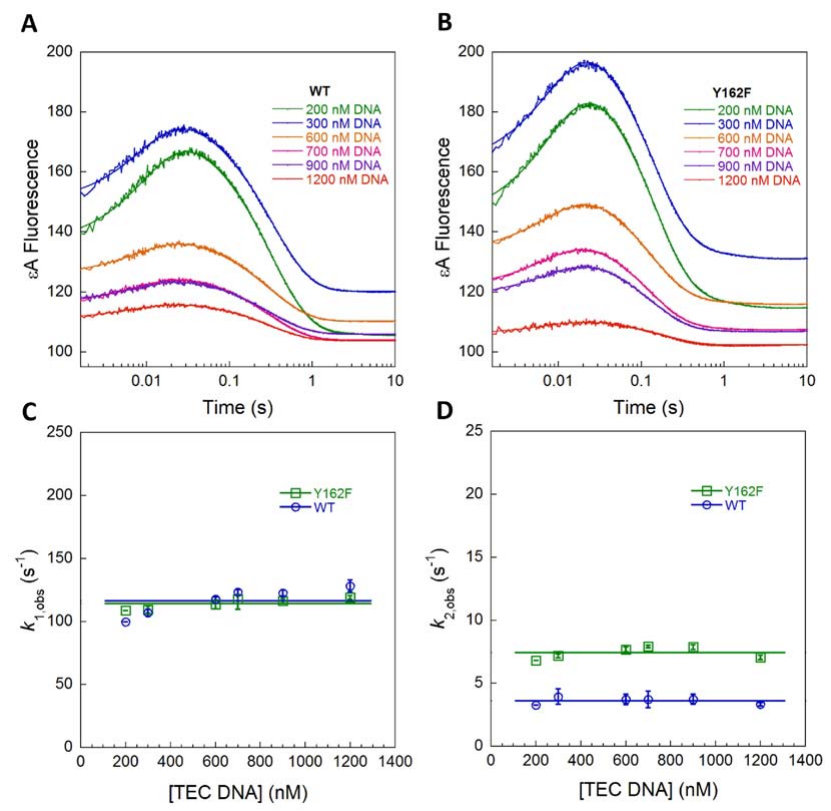
Stopped-flow fluorescence with excess DNA to measure *k*_find_ for WT and Y162F AAG. Conditions of excess DNA ensure that binding events involve only a single protein. Representative data from experiments in which 100 nM of WT (**A**) or Y162F (**B**) protein was mixed with increasing concentrations of TEC DNA. The εA-fluorescence was collected and fit by Equation (S4) as described for Figure 3. (**C**) With excess DNA, the rate constant for the first phase (*k*_1,obs_) is independent of concentration and is designated *k*_find_. (**D**) The rate constant for the second phase (*k*_2,obs_) is also concentration independent and corresponds to nucleotide flipping. This value is identical to that measured in stopped-flow experiments with excess protein (Table 1). Rate constants in (C) and (D) are from three independent experiments (average ± SD).

We previously noted that the initial recognition complex, in which the εA lesion is partially unstacked, appears to form with the bimolecular rate constant for association (11). As AAG uses facilitated diffusion to search for sites of damage (13), the searching steps must be faster than the association with nonspecific DNA (Figure 1C). We hypothesized that this fast searching time is due to multiple proteins simultaneously searching for the εA site under conditions of excess protein, and the searching time for a single protein would be significantly slower. Therefore, we also performed stopped-flow association experiments in which εA-DNA was in excess over WT (Figure 4A) or Y162F AAG (Figure 4B). Although the fraction of DNA that is bound is much smaller under these conditions, there is sufficient signal to accurately measure protein binding. As was observed for conditions of excess protein, the individual fluorescence traces showed biphasic increase and decrease in fluorescence, but now the first observed rate constant was independent of concentration (Figure 4C). We designate this observed rate constant as *k*_find_, the macroscopic rate constant for the rapidly reversible searching steps that culminate in the formation of the initial recognition complex (Figure 1C). The Y162F mutation did not alter the searching process significantly (Figure 4C; *k*_find_ ≈ 115 s^−1^ for both WT and Y162F AAG). The observed rate constant for the formation of the flipped-out specific recognition complex when there was excess DNA was the same within error as when there was excess protein (Figure 4D; Table 1). This indicates that excess protein does not interfere with nucleotide flipping.

**Table 1.** Kinetic parameters for recognition and excision of εA^a^

	WT	Y162F	Y162A
*k*_on_ (M^−1^s^−1^)	(1.1 ± 0.03) x 10^9^	(2.1 ± 0.2) x 10^9^	Fast^d^
*k*_find_ (s^−1^)	116 ± 11	114 ± 4	Fast^d^
*k*_flip_ (s^−1^)	3.6 ± 0.7 (3.6 ± 0.3)^c^	7.9 ± 1.0 (7.4 ± 0.5)^c^	170 ± 16^e^
*k*_unflip_ (s^−1^)	(1.6 ± 0.3) x 10^−3^	(4.6 ± 0.2) x 10^−3^	10 ± 1^f^
*K*_flip_^b^	2300	1700	17^f^
*k*_chem_ (s^−1^)	(8.0 ± 0.6) x 10^−4^	(4.3 ± 0.2) x 10^−4^	(3.8 ± 0.1) x 10^−4^

^a^Rate constants were determined from changes in εA fluorescence or glycosylase activity using the TEC oligonucleotide, unless otherwise indicated. The standard conditions were 25°C, 50 mM NaMES, pH 6.5, 100 mM NaCl, 1 mM EDTA, 1 mM DTT.

^b^The equilibrium constant for flipping is given by the ratio of the flipping and unflipping rate constants (*K*_flip_ = *k*_flip_/*k*_unflip_).

^c^Values from stopped-flow experiments with excess protein (values with excess DNA are in parenthesis).

^d^*k*_on_ and *k*_find_ are too fast to measure for Y162A.

^e^A value of 66 ± 2 s^−1^ was determined for the AEA oligonucleotide with excess protein (Figure 5D).

^f^The AEA oligonucleotide was used to measure dissociation, because it gives a larger change in fluorescence (Figure 6B). Only a limit could be obtained for the dissociation of the TEC oligonucleotide (Figure 6A); therefore, the values shown are estimates based on the dissociation of the AEA oligonucleotide. For comparison, the equilibrium constant determined for flipping of the AEA oligonucleotide by Y162A is ∼7 (*K*_flip_ = 66/10).

**Figure 5.**
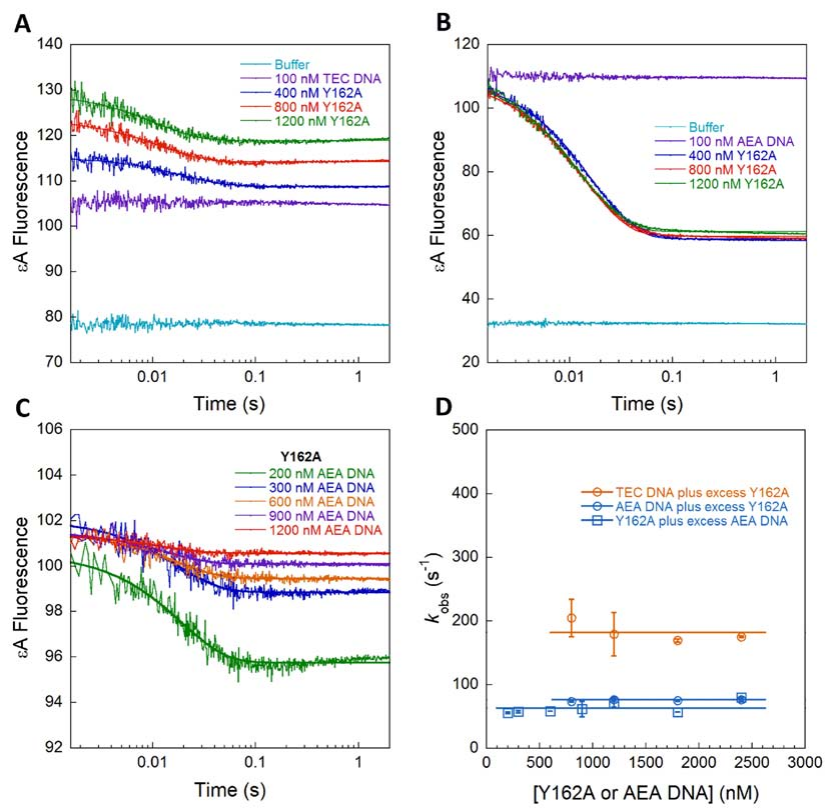
Stopped-flow fluorescence to measure nucleotide flipping by Y162A AAG. Representative reactions in which 100 nM of TEC (**A**) or AEA (**B**) εA-DNA was mixed with increasing concentrations of excess protein. (**C**) Representative data for binding of 100 nM Y162A AAG with increasing concentrations of AEA DNA. In each case, the changes in fluorescence were fit by a single exponential. (**D**) Observed rate constants (*k*_obs_ = *k*_flip_ + *k*_unflip_) from three independent experiments are plotted as a function of concentration (average ± SD). The initial binding and the formation of the initial recognition complex are not observed, indicating that they are both faster than the nucleotide flipping step.

Association experiments with Y162A AAG yielded very different fluorescence traces. When excess Y162A AAG was mixed with εA-DNA (TEC), only a single time-dependent decrease in fluorescence was observed (Figure 5A). Even at low concentrations of protein, the first phase of the reaction was complete in the dead time of the stopped-flow (*k*_on_ ≥ 10^9^ M^−1^s^−1^). The exponential decrease in fluorescence, which indicates formation of the flipped-out specific recognition complex, was independent of concentration and much faster than either the WT or Y162F AAG (Figure 5D, orange symbols). The changes in fluorescence were quite small for the TEC DNA. Therefore, we also measured association kinetics for the AEA DNA with excess protein (Figure 5B) and with excess DNA (Figure 5C). For the Y162A mutant protein, almost identical rate constants were observed whether protein or DNA was in excess (Figure 5D, blue symbols). These data indicate that Y162A AAG associates with nonspecific DNA and searches significantly faster than the WT enzyme, such that the searching step is not rate-limiting under any of the conditions tested. Remarkably, the observed flipping step is also significantly faster for the Y162A mutant than for WT AAG (50-fold faster for the TEC DNA; Table 1).

### Measurement of unflipping and dissociation of εA-DNA

**Figure 6.**
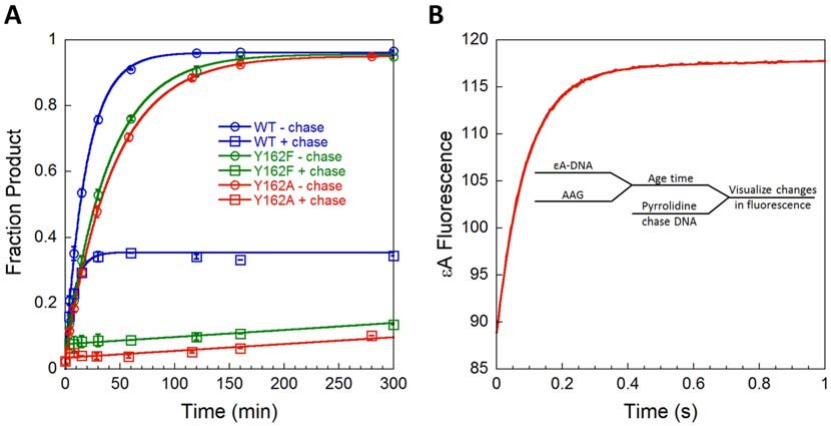
Pulse-chase experiments to measure dissociation of εA-DNA. (**A**) The partitioning between the forward reaction (base excision) and substrate dissociation was measured using a gel-based assay. The commitment for WT (30%), Y126F (8%) and Y162A (0%) were used to calculate the rates of unflipping (see Methods). The low commitment for Y162F AAG was reproducible and readily distinguished from the control in which chase and substrate are added at the same time (Supplementary Figure S2). (**B**) Double-mixing experiments were performed using stopped-flow fluorescence to monitor the increase in fluorescence upon release of εA-DNA. The AAG·DNA complex was formed, aged for 1 s, and then chased with an excess of pyrrolidine-DNA as a competitor. The data were fit by a single exponential and the average ± SD from three independent experiments is included in Table 1.

As the binding and nucleotide flipping steps are reversible, the observed formation of the specific complex is affected by both forward and reverse rates. Therefore, it is necessary to measure the dissociation kinetics to define individual microscopic rate and equilibrium constants. The dissociation of AAG from εA-containing DNA was investigated using the pulse-chase method, whereby the AAG·εA-DNA complex is allowed to form and is subsequently chased with an excess of tight binding pyrrolidine-DNA inhibitor (29). Any AAG that dissociates is bound to the inhibitor, allowing measurement of the partitioning forward (abasic DNA product) and backward (εA-DNA substrate) as previously described (11,12). For WT AAG, 30% of the complex partitions forward to produce product and 70% dissociates (Figure 6A and Supplementary Figure S2). Assuming that dissociation of AAG from nonspecific DNA is fast, these data yield the microscopic rate constant for unflipping [Equation (S6)]. More complete analysis is consistent with fast dissociation from nonspecific DNA (Supplementary Figure S3). The Y162F mutant showed greater dissociation of the substrate (90% dissociated; Figure 6A), indicating a 3-fold faster value of *k*_unflip_ compared to WT (Table 1). In contrast, 100% of the Y162A mutant dissociated and no product could be detected in the presence of chase (Figure 6A), indicating that unflipping is much faster than *N*-glycosidic bond cleavage. In order to precisely measure the rate of unflipping for the Y162A mutant, we next performed a double-mixing experiment in the stopped-flow.

In the double-mixing experiment, the flipped-out complex of Y162A AAG with εA-DNA was formed and then challenged with pyrrolidine-DNA competitor (Figure 6B; inset scheme). The time-dependent increase in εA fluorescence is fit by a single exponential, which corresponds to the rate-limiting unflipping step. This experiment reveals that the rate of εA unflipping for Y162A AAG (*k*_unflip_ = 10.3 s^−1^) is 6000-fold faster than that of WT AAG (Table 1). Although the Y162A mutation causes only a modest defect in glycosylase activity toward εA, it imparts a dramatic effect on nucleotide unflipping.

With the rate constants for unflipping in hand, the rate and equilibrium constants for nucleotide flipping could be calculated for WT and mutant enzymes (Table 1). The microscopic rate constant for flipping was obtained from the observed rate constant for formation of the extrahelical complex (*k*_flip_ = *k*_2,obs_ − *k*_unflip_), and the equilibrium constant for nucleotide flipping is the ratio of the two values (*K*_flip_ = *k*_flip_/*k*_unflip_). The Y162F mutant exhibits a *K*_flip_ value that is almost identical to WT AAG, with 2-fold faster rates for both flipping and unflipping. In contrast, the Y162A mutant has a greatly reduced equilibrium constant for flipping that is 140-fold less favorable than that of the WT enzyme. The destabilization of the specific recognition complex is caused by a 6000-fold increased rate of unflipping that is only partially balanced by a 50-fold increased rate of flipping (Table 1).

### Competition between εA-DNA and undamaged DNA

**Figure 7.**
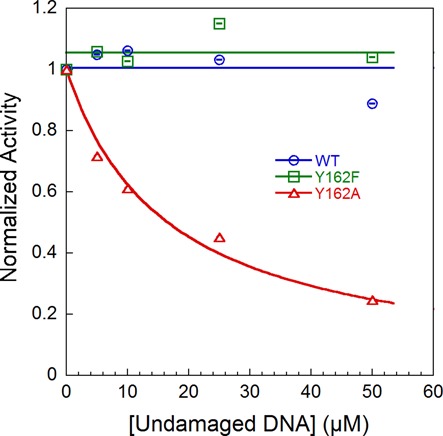
Competition between εA-DNA and undamaged DNA. Single-turnover excision of εA by WT and mutant AAG was measured as described in Figure 2B with 200 nM εA-DNA, 300 nM enzyme and the indicated concentration of undamaged DNA. The observed rate constants were normalized by dividing by the rate constant in the absence of competitor (average ± SD; three independent experiments). WT and Y162F AAG are unaffected by the presence of excess undamaged DNA. In contrast, Y162A AAG is inhibited by the addition of competitor DNA (IC_50_ = 20 μM).

Although Y162A exhibits only a modest decrease in single-turnover glycosylase activity, the decreased stability of the flipped-out complex suggests that this mutant would be unable to efficiently find sites of εA damage when sites of damage are rare. We tested this hypothesis by using undamaged DNA as a competitor. Under single-turnover conditions, with enzyme in excess over the εA-labeled DNA substrate, the single-turnover rate constant for εA excision by WT and Y162F AAG was unchanged in the presence of excess undamaged DNA (Figure 7). In contrast, Y162A AAG is inhibited by undamaged DNA with an IC_50_ value of 20 μM. This concentration of undamaged DNA corresponds to 5000-fold excess of undamaged over damaged nucleotides, which is far below the expected ratio of undamaged to damaged sites in the human genome. Thus, the Y162A mutant is much less efficient than the WT protein at repairing sites of damage when sites of damage are rare. This could explain why the Y162A mutant is less effective than WT AAG at protecting yeast from DNA alkylation (14).

## DISCUSSION

Structures of DNA repair glycosylases in complex with DNA suggest that nucleotide flipping is aided by intercalating residues (3,19–20,24). The prevalence of nucleotide flipping and the dramatic nature of the conformational change that accompanies DNA bending and nucleotide flipping have captured the interest of biologists and chemists alike. However, little is known about the fundamental mechanisms by which nucleotide flipping is accomplished because it is difficult to observe rapid conformational changes that occur on the millisecond time scale. To understand the contribution of DNA intercalation to the energetic landscape for specific recognition and nucleotide flipping, we performed comprehensive kinetic analysis of mutant forms of AAG in which the conserved intercalating tyrosine (Y162) was mutated. These results provide support for the model that intercalation by an aromatic side chain greatly stabilizes the extrahelical conformation of the specific recognition complex. Unexpectedly, we further identify a role for Y162 in slowing the rate of nucleotide flipping, apparently by stabilizing the initial recognition complex. The new model that emerges suggests that DNA intercalation contributes to the specificity of AAG, but the increased specificity comes at the cost of reduced speed. These results provide new insight into the mechanism of AAG, and have implications for other enzymes that use nucleotide flipping for DNA repair or epigenetic DNA modification.

### Kinetic mechanism of AAG

Since crystal structures cannot capture the dynamic nature of DNA glycosylases, spectroscopic assays are needed to elucidate reaction mechanisms. Previous work found that AAG and other DNA glycosylases use multistep recognition to distinguish damaged sites from among an excess of undamaged sites (11–12,20,30). We performed a series of transient kinetic experiments to dissect the individual steps of binding, searching, flipping and base excision by AAG (Figure 1C). The individual microscopic rate constants were calculated from the association and dissociation experiments and summarized in Table 1. In addition, we performed a global fitting of this kinetic mechanism to the data for association reactions with excess protein and excess DNA that is detailed in the Supplementary Materials (Supplementary Figures S3–S6). Overall the two approaches yielded similar microscopic rate constants (Supplementary Figures S3E and S4D). Our results with the WT enzyme are in agreement with previously reported values (11), but we have gained new insight into the DNA searching process by directly measuring the rate of searching under conditions of excess DNA.

By employing a DNA sequence context in which the εA lesion is quenched (TEC), we observed a transient intermediate in which the εA is less strongly quenched and presumably partially unstacked (Figure 3A). Although binding of AAG to the εA lesion appears to be a bimolecular reaction (Figure 3C), we favor the model that nonspecific DNA binding and rapid facilitated diffusion allow AAG to locate and bind the damaged nucleotide. Under the conditions of excess protein that are most commonly employed to monitor labeled DNA, simultaneous searching by multiple AAG molecules makes this searching step too fast to detect (11). We have modeled searching by multiple proteins explicitly to show that an additive model, in which two proteins search twice as fast as one protein, is consistent with the experimental data (Supplementary Figure S3). To directly measure the searching by a single molecule of AAG, we employed conditions of excess DNA (Figure 4). Under these conditions, the initial recognition complex is formed with an observed unimolecular rate constant of 120 s^−1^ (*k*_find_; Table 1), which is equal to the sum of the rate constants for finding and leaving the site of damage. For WT AAG, this initial recognition complex is significantly populated, indicating that the rate of finding the εA site is faster than the rate of leaving the site.

Although multiple AAG molecules can bind to a single oligonucleotide, the observed rate constant for nucleotide flipping is identical under conditions of excess DNA or excess protein (Table 1). This indicates that multiple proteins do not affect the flipping step and that rate constants obtained in experiments with multiple or single proteins bound can be directly compared. To define the microscopic rate constants for flipping and the equilibrium constant for flipping, the dissociation of the bound εA-DNA must also be measured. Our results demonstrate that WT AAG has a very slow rate of unflipping and an extremely favorable equilibrium constant for stabilizing the flipped-out nucleotide (Table 1). We tested the specificity of AAG by employing conditions with 12 500-fold excess of undamaged sites (50 μM undamaged oligonucleotide) and found that the observed rate of εA excision was unaffected (Figure 7). Thus, AAG is able to compensate for slow *N*-glycosidic bond cleavage by binding very tightly to εA-DNA.

### Contributions of DNA intercalation by Tyr 162

We focused on the role of the intercalating tyrosine, which is universally conserved among eukaryotic AAG homologs (19,31). We find that both the Y162F and Y162A mutations are well tolerated *in vitro* and do not appear to greatly perturb the structure, because the rate constant for *N*-glycosidic bond cleavage is within 2-fold of WT AAG. Therefore, the comparison of WT and mutant proteins provides valuable insights into the functional contributions of tyrosine to the individual microscopic steps in the recognition of εA lesions.

The kinetic parameters for the Y162F variant are almost identical to those of the WT protein (Table 1). Association, flipping and unflipping are approximately 2-fold faster for this mutant, whereas the searching rate constant and the overall equilibrium constant for flipping are essentially identical to WT AAG. This indicates that the hydroxyl group of tyrosine is not necessary for any of the microscopic steps associated with finding and flipping out εA lesions. Some prokaryotic homologs of AAG have been identified that appear to substitute histidine for tyrosine at the intercalating position (19,32). It is easy to imagine that histidine could make similar interactions as tyrosine and phenylalanine. Interestingly, Y162 has been identified as the primary site of nitration, which is reported to decrease glycosylase activity (27). It is possible that nitration of tyrosine serves a biological function that could account for the conservation of this residue. Future *in vivo* experiments with the Y162F mutant, that cannot be nitrated, may be able to shed light on this issue (27).

The comparison of Y162A to WT AAG allows the energetic contribution of DNA intercalation to be measured for individual steps in the kinetic mechanism. Both association and searching are significantly faster for Y162A, but were too fast to measure with stopped-flow assays. Although the Y162A mutant engages εA sites significantly faster than the WT enzyme, it cannot plug the vacated space left by the extrahelical lesion and lacks the favorable pi–pi stacking interactions made by the aromatic side chain. In the absence of this stacking energy, the specific recognition complex is greatly destabilized. The faster flipping exhibited by the Y162A mutant argues against a pushing model, and suggests instead that the aromatic side chain of Y162 helps to stabilize a partially unstacked initial recognition complex. This mutant is not able to effectively find sites of damage when challenged by an excess of undamaged bases (Figure 7), leading to the prediction that DNA intercalation is essential for *in vivo* DNA repair. Consistent with this conclusion, the Y162A mutant was found to be less efficient than WT AAG at protecting yeast cells from exposure to an exogenous DNA alkylating agent (14).

Parallels can be drawn between the consequences of the Y162A mutation in AAG and the results that have been obtained for other families of DNA repair glycosylases. For example, it has been reported that mutation of intercalating residues to alanine in other DNA glycosylases increases the rate of diffusion on DNA, presumably by destabilizing searching complexes (33,34). This suggests a common theme of DNA intercalation as an integral component of the more extensive interactions that distinguish damaged from undamaged sites, and the sampling of these conformations is time consuming.

### Conclusions

Structures of AAG bound to εA-DNA (19) raised the possibility that intercalation by Y162 could increase the rate constant for flipping by ‘pushing’ or destabilizing the duplex and decrease the rate constant for unflipping by ‘plugging’ the hole vacated by the flipped-out nucleobase, as has also been suggested for other glycosylases (20,24). Our study provides a rigorous test of these models. The kinetic parameters for Y162A support the plugging model, with this mutant showing a 6000-fold increase in the rate of unflipping relative to WT AAG. Surprisingly, the Y162A mutant does not show a reduced rate of flipping, but instead it flips out εA lesions 50-fold faster than the WT enzyme. This argues against the pushing model for Y162 and demonstrates that intercalation of Y162 dramatically slows the nucleotide flipping step. The slower rate constant for flipping by WT AAG is presumably explained by tight binding of εA in an initial recognition complex. Consistent with this view, the initial recognition complex does not appear to stably form for the Y162A mutant (Figure 5). Overall, the Y162A mutation greatly destabilizes the specific recognition complex, and we obtain an estimate of 140-fold stabilization for the contribution of tyrosine intercalation to the equilibrium constant for flipping. AAG is a particularly attractive system to delve into the biophysical and biochemical mechanism of multistep DNA recognition, but it is apparent that many different enzymes must employ similar strategies to discriminate between nonspecific and specific sites. The lessons learned from AAG may be more widely applicable to other DNA-modifying enzymes, and it will be interesting to learn to what extent independently evolved enzymes rely on these specific strategies for locating and gaining access to nucleotide targets in duplex DNA.

## SUPPLEMENTARY DATA

Supplementary Data are available at NAR Online.

SUPPLEMENTARY DATA
