# Supplementary material for: Critical role of DNA intercalation in enzyme-catalyzed nucleotide flipping
Source: Nucleic Acids Res. 2014 Oct 16;42(20):12681–90. doi: 10.1093/nar/gku919 (PMC4227769; doi:10.1093/nar/gku919)
Supplement: SUPPLEMENTARY DATA [file supp_42_20_12681__index.html]

Critical role of DNA intercalation in enzyme-catalyzed nucleotide flipping — Critical role of DNA intercalation in enzyme-catalyzed nucleotide flipping — SUPPLEMENTARY DATA 

# Critical role of DNA intercalation in enzyme-catalyzed nucleotide flipping

## SUPPLEMENTARY DATA

**Files in this Data Supplement:**

- SUPPLEMENTARY DATA
