# Supplementary material for: Critical role of DNA intercalation in enzyme-catalyzed nucleotide flipping
Source: Nucleic Acids Res. 2014 Oct 16;42(20):12681–90. doi: 10.1093/nar/gku919 (PMC4227769; doi:10.1093/nar/gku919)
Supplement: SUPPLEMENTARY DATA [file supp_gku919_nar-01845-f-2014-File009.pdf]

Supplementary Material

"Critical role of DNA intercalation in enzyme-catalyzed nucleotide flipping"

Jenna M. Hendershot and Patrick J. O'Brien*

University of Michigan, Department of Biological Chemistry, Ann Arbor MI 48109-0600

*To whom correspondence should be addressed: Department of Biological Chemistry, University of Michigan Medical School, 5301 MSRB III 1150 W Medical Center Drive, SPC 5606 Ann Arbor MI 48109. Phone: 734-647-5821 Fax: 734-763-4581 Email: pjobrien@umich.edu.

Contents of Supplementary Material:

Supplementary methods pg. S2 – S5

S1. Single-turnover excision of εA for WT, Y162F, and Y162A AAG pg. S6

S2. Complete data for pulse-chase assays, including chase controls pg. S7

S3. Global fits to stopped-flow experiments with excess protein pg. S8

S4. Global fits to stopped-flow experiments with excess DNA pg. S9

S5. Berkeley Madonna script for global fitting of excess protein pg. S10 – S11

S6. Berkeley Madonna script for global fitting of excess DNA pg. S12 – S13

Supplementary references pg. 14

Supplementary methods

Purification of WT and Mutant AAG Proteins. The catalytic domain of human AAG that lacks the first 79 amino acids was expressed in *E. coli* and purified as previously described (1). The Y162F and Y162A mutants were constructed by site-directed mutagenesis and purified using the same protocol. The concentration of AAG was determined from the UV absorbance and predicted extinction coefficient. In the case of Y162F and WT proteins, burst experiments were used as previously described to determine the active concentration (2). Using this method, WT was found to be 86% active and the Y162F was found to be 42% active. The corrected concentration was used for all of the experiments described below. These concentrations were independently validated by titration with εA-DNA (Figure 2A in the main text), as WT, Y162A, and Y162F AAG all showed the expected equivalence point corresponding to 1:1 binding of the same DNA stock.

Synthesis and Purification of Oligodeoxynucleotides. Oligonucleotides were synthesized by Integrated DNA Technologies or the W.M. Keck Facility at Yale University, were purified and the concentrations determined from the calculated extinction coefficients as previously described (2). Duplexes were annealed with a 1.2-fold excess of the unmodified complement by heating to 90°C and cooling slowly to 4 ^{o}C.

Steady-State Fluorescence Measurements. Fluorescence emission spectra were collected with a PTI QuantaMaster fluorometer controlled by FeliX software. For εA fluorescence, an excitation wavelength of 314 nm (6 nm band-pass) was used and the total fluorescence was measured at emission wavelengths from 340-480 nm (6 nm band-pass). Samples (300 µL) of 400 nM εA-containing DNA were prepared in the standard buffer [50 mM NaMES (pH 6.5), 100 mM NaCl, 1 mM EDTA, 1 mM DTT] and spectra were recorded at 25 ^{o}C. To determine the steady-state fluorescence of εA-containing DNA bound to AAG, the spectra were recorded within 1 minute to ensure that no significant excision of εA occurred. Four independent titrations were averaged, and the data were fit by a quadratic equation assuming tight binding by AAG (Eq. 1), in which F_{rel} is the relative fluorescence, A is the fractional quenching, and K_d is the dissociation constant for the εA-DNA.

$$F_{rel} = 1 - \frac{A(K_d + E + DNA) - \sqrt{(K_d + E + DNA)^2 - (4E \times DNA)}}{2E} \qquad (1)$$

Gel-based Glycosylase Assay. Single-turnover glycosylase activity was measured with a discontinuous assay that utilizes abasic site cleavage by NaOH followed by DNA separation on a denaturing polyacrylamide gel (3). Fluorescein-labeled (5'-6-fam) DNA substrates (50 nM) containing εA were prepared in the standard buffer. Reactions were initiated with the addition of 75 nM – 600 nM AAG and incubated at 25 ^{o}C. At various time points, a sample from the reaction was removed and quenched in 2 volumes of 0.3 M NaOH, giving a final hydroxide concentration of 0.2 M. The abasic sites were cleaved by heating at 70 ^{o}C for 15 minutes. Samples were mixed with an equal volume of formamide/EDTA loading buffer before loading

onto a 15% polyacrylamide gel. Gels were scanned with a Typhoon Imager (GE Trio+ Healthcare) to detect the fluorescein label by exciting at 488 nm and measuring emission with a 520BP40 filter. The gel bands were quantified using ImageQuant TL (GE Healthcare). The data were converted to fraction product [Fraction Product = product / (product + substrate)] and then fit by a single exponential using Kaleidagraph (Synergy Software) (Eq. 2). Single-turnover rates were independent of the concentration of AAG, indicating that the maximal rate constant was measured ($k_{obs} = k_{max}$; Fig. S1).

$$\text{Fraction product} = A(1 - e^{-k_{obs}t}) \qquad (2)$$

To measure competition between εA-DNA and undamaged DNA, single-turnover reactions containing 200 nM fluorescein-labeled εA DNA and 0–50 μM undamaged DNA, in which the εA was replaced by a normal A, were initiated with the addition of 300 nM AAG. Data for Y162A AAG was fit by an IC_{50} equation (Eq. 3); WT and Y162F AAG were fit by a line with a slope of zero.

$$\text{Activity} = \frac{1}{1 + [I]/IC_{50}} \qquad (3)$$

Stopped-Flow Kinetics. Pre-steady state kinetic experiments were performed on a Hi-Tech SF-61DSX2, controlled by Kinetic Studio (TgK Scientific). The fluorescence of εA was monitored using an excitation wavelength of 313 nm and a WG360 long-pass emission filter (3, 4). At least three traces were averaged together at each concentration. We found that the fluorescence amplitudes varied from day to day with different instrument settings, but that the observed rate constants were highly reproducible. Therefore, we allowed the amplitudes to float in the curve fitting with Kaleidagraph. These amplitudes were not used in the data analysis. The traces for WT and Y162F AAG were fit by a triple exponential (Eq. 4) where *F* is the fluorescence as a function of time, *C* is the fluorescence of free DNA, *X, Y,* and *Z* are the changes in fluorescence of the intermediates, and *t* is the time. The traces for Y162A AAG were fit by a single exponential (Eq. 2) and k_{obs} is equal to $k_{flip} + k_{unflip}$.

$$F = C - X(1 - e^{-k_{1,obs}t}) - Y(1 - e^{-k_{2,obs}t}) - Z(1 - e^{-k_{3,obs}t}) \qquad (4)$$

Observed rate constants were plotted versus concentration and fit by a straight line. $k_{1,obs}$ showed a linear concentration dependence, and the slope is equal to k_{on} ($M^{-1}s^{-1}$). Negative y-intercepts were observed under these conditions. Similar negative intercepts were reported for another DNA binding protein with high affinity for DNA (5). The value of $k_{2,obs}$ was essentially independent of concentration and is equal to $k_{flip} + k_{unflip}$. It should be noted that in some cases the lowest concentrations gave slightly lower values of $k_{2,obs}$. The kinetic scheme predicts that a hyperbolic dependence would be observed at lower concentrations, as the initial recognition complex becomes saturated. However, there was not sufficient fluorescence signal to explore this region of the concentration dependence. Fitting of the data to hyperbolic fits gave

essentially identical maximal values of $k_{2,obs}$, confirming that the initial recognition complex was saturated by the highest concentrations of DNA and protein that were tested (data not shown).

As an independent test of the kinetic parameters that were determined by combining association and dissociation kinetics, we fit the observed association kinetics obtained with either excess protein or excess DNA with the kinetic model using Berkeley Madonna (Berkeley Madonna, Inc.). The analysis with excess protein is given in Figure S3, according to the mechanism and script provided in Figure S5. Note that we must explicitly consider multiple bound proteins to explain the differences between excess protein and excess DNA. The analysis with excess DNA is given in Figure S4, according to the mechanism and script provided in Figure S6.

Pulse-Chase Assay to Measure Substrate Dissociation. The macroscopic rate constant for dissociation of WT and mutant AAG from εA-containing DNA was measured by pulse-chase in the standard reaction buffer at 25 ^{o}C as previously described for WT AAG (3, 4). In 20 µL reactions, 50 nM fluorescein-labeled TEC DNA was mixed with 300 nM or 600 nM AAG for 20 seconds, and then a chase of 10 µM pyrrolidine-containing DNA was added. Pyrrolidine binds tightly to AAG as a transition state analogue (6, 7). At various time points, a sample from the reaction was removed to evaluate the partitioning of a bound E•S complex between *N*-glycosidic bond hydrolysis and dissociation with the standard glycosylase assay. The fraction of product was fit by a single exponential (Eq. 2). Control reactions in which no chase was added provided the single-turnover rate constant, k_{max}, and confirmed that these concentrations of AAG were saturating. From these values, the dissociation rate constant, k_{off}, for AAG dissociating from εA-DNA was calculated as previously described. The rearranged equation is indicated by Eq. 5, in which A is the burst amplitude (the fraction of product formed in the burst phase of the experiment), k_{max} is the single-turnover rate constant for formation of product, and $k_{off,obs}$ is the macroscopic rate constant for dissociation from the flipped-out complex.

$$k_{off,obs} = \frac{k_{max}}{A} - k_{max} \qquad (5)$$

Since the εA-DNA binds in two steps, the observed rate constant for dissociation of substrate ($k_{off,\ obs}$) could be limited by the unflipping rate (k_{unflip}) or dissociation from nonspecific DNA (k_{off}). According to Fig. 1C, and assuming that the flipped-out complex is stable (i.e., $k_{flip} >> k_{unflip}$), this observed dissociation rate constant can be expressed in terms of the microscopic rate constants [Eq. 6; (8)]. Given rapid dissociation from nonspecific DNA, the observed rate of dissociation from the εA-DNA•AAG complex is approximately equal to the reverse rate constant for flipping ($k_{off,obs} \approx k_{unflip}$).

$$k_{off,obs} = k_{unflip}\left(\frac{k_{off}}{k_{off} + k_{flip}}\right) \qquad (6)$$

Direct measurement of substrate dissociation. Stopped-flow double-mixing experiments were performed to measure unflipping and dissociation of εA-DNA by Y162A. To rapidly form the

flipped-out AAG-DNA complex, 2.8 μM AEA DNA was mixed with 2 μM Y162A. After an aging time of 1 second, 18 μM or 36 μM pyrrolidine-DNA was added as a competitor. The final concentrations after mixing were 700 nM AEA DNA, 500 nM Y162A, and 9 μM or 18 μM pyrrolidine-DNA. The reaction was followed for 10 seconds; no significant excision of εA occurs during this time. Three traces were averaged together and the change in fluorescence was fit by a single exponential (Eq. 2). As described for the pulse-chase assay, the observed rate constant for dissociation is equal to the rate constant for unflipping.

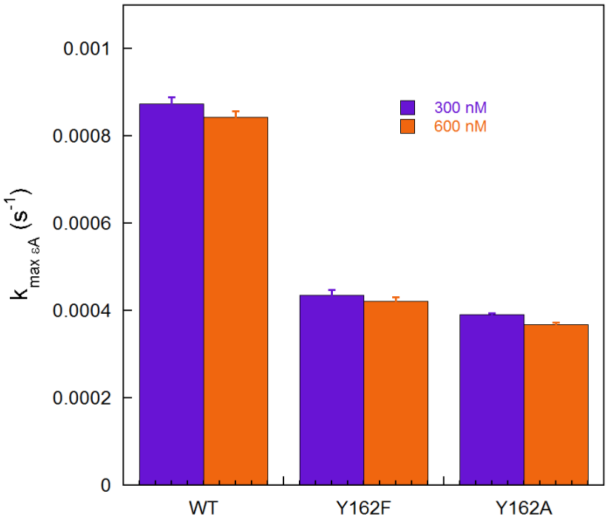

Figure S1. Single-turnover excision of εA for WT and mutant AAG. Essentially identical rate constants were obtained for excision of εA from TEC DNA (50 nM) and either 300 nM or 600 nM AAG. These data indicate that 300 nM enzyme is saturating for all of the AAG variants tested.

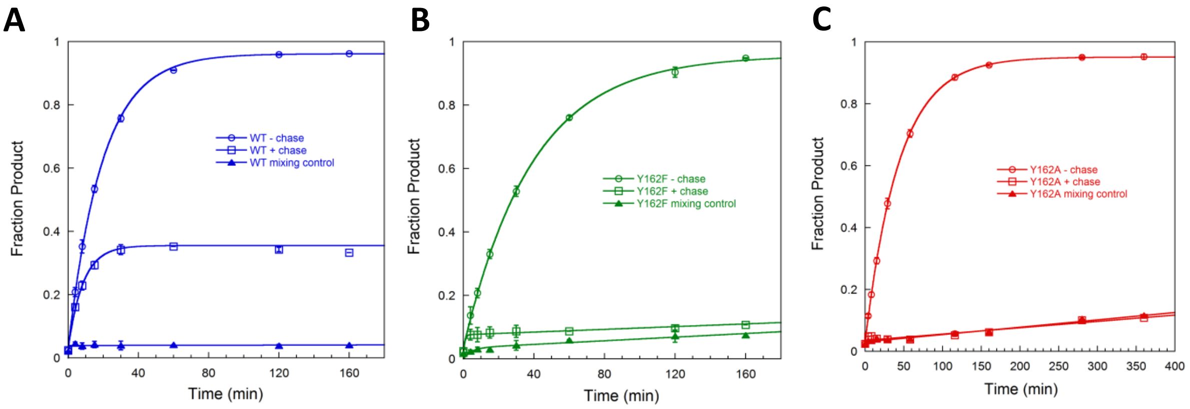

Figure S2. Pulse-chase experiment for measuring the partitioning between excision and dissociation of εA-DNA for WT (A), Y162F (B), and Y162A (C) AAG. As a control to test the effectiveness of the unlabeled pyrrolidine-DNA chase, reactions were initiated by adding AAG to a mixture of chase and 5'FAM εA-DNA substrate (triangles). The reactions in the presence (squares) and absence (circles) of chase are from Fig. 6 in the text. Comparison of the +chase reaction and the chase control for Y162F supports a small, but measurable commitment to excision.

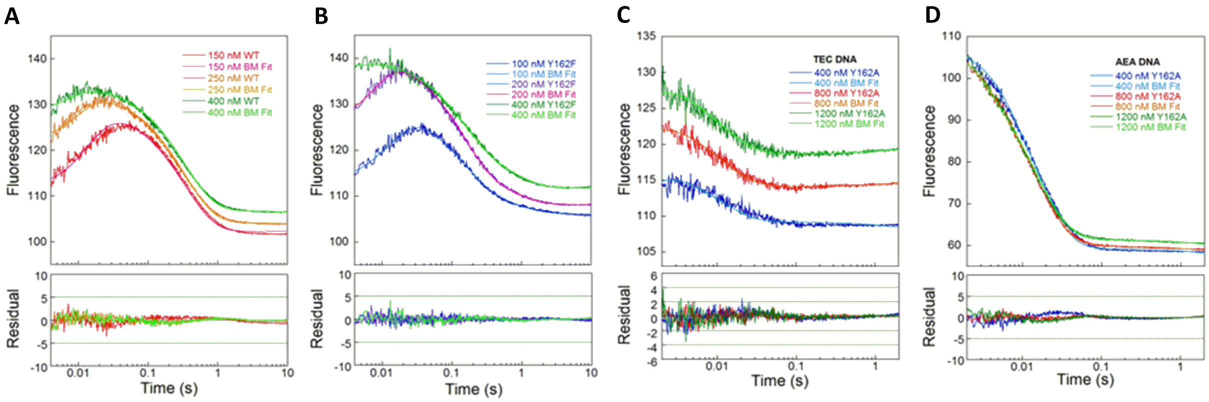

E

Excess Protein	Experimental WT (TEC)	BM WT	Experimental Y162F (TEC)	BM Y162F	Experimental Y162A (TEC)	BM Y162A	Experimental Y162A (AEA)	BM Y162A
$k_1 = k_{on}$	1.1×10^{9}	1.3×10^{9}	2.1×10^{9}	2.4×10^{9}	n/a	1.8×10^{10}	n/a	1.6×10^{9}
$k_2 = k_{off}$	n/a	41.4	n/a	62.9	n/a	12.9	n/a	63.4
k_3	n/a	84.2	n/a	93.7	n/a	226.9	n/a	148.5
k_4	n/a	31.3	n/a	15.7	n/a	693.5	n/a	67.6
$k_{find} = k_3 + k_4$	116	115.5	114	109.4	n/a	920.4	n/a	216.1
$k_7 = k_{flip}$	3.6	3.3	7.7	7.5	171.8	171.0	66.0	66.3
$k_8 = k_{unflip}$	0.002	0.0012	0.005	0.005	10.3	10.3	10.3	10.4
$k_9 = k_{artifact}$	0.51 – 1.72	0.012	0.66 – 2.14	1.3	0.03 – 1.8	16.6	1.22 – 12.9	6.5

Figure S3. Representative Berkeley Madonna (BM) global fits to excess protein stopped-flow experiments (A) WT (B) Y162F (C and D) Y162A AAG. Lines show the best fits obtained with Berkeley Madonna (see S5), and the plot below shows the residual between the fit and the experimental data. (E) Table summarizing experimental values from the individual fits (taken from Table 1 in main text) and BM (Berkeley Madonna) global analysis of curves shown in A–D.

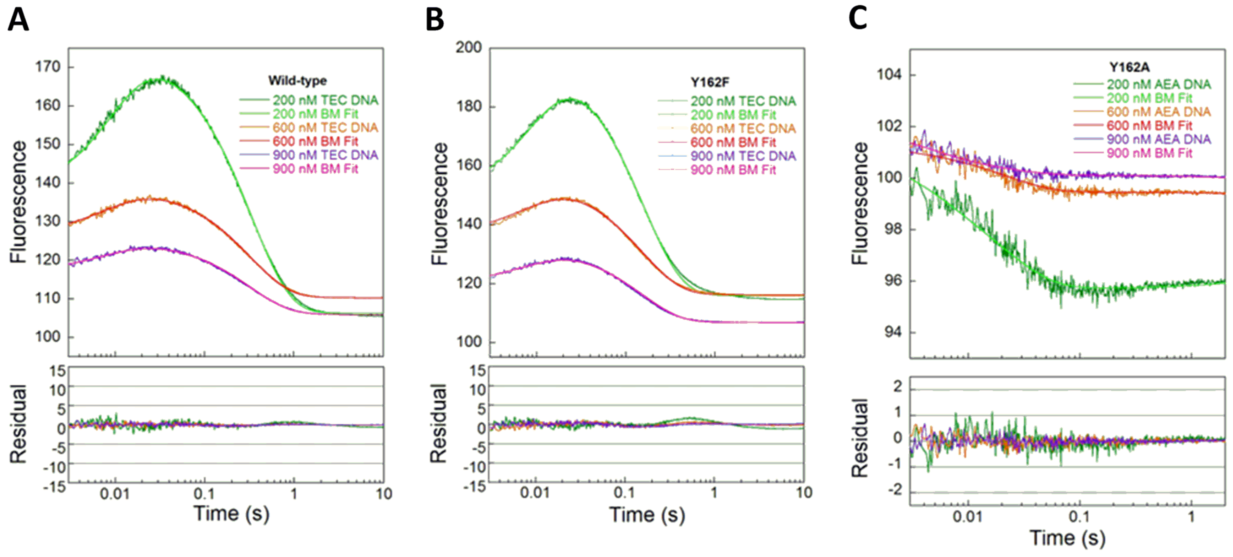

D

Excess DNA	Experimental WT	BM WT (TEC)	Experimental Y162F	BM Y162F (TEC)	Experimental Y162A	BM Y162A (AEA)
$k_1 = k_{on}$	1.1×10^9	3.8×10^9	2.1×10^9	4.3×10^9	n/a	1.2×10^{10}
$k_2 = k_{off}$	n/a	52.9	n/a	62.3	n/a	65.2
k_3	n/a	104.9	n/a	102.6	n/a	173.4
k_4	n/a	18.3	n/a	16.8	n/a	56.2
$k_{find} = k_3 + k_4$	116	123.2	114	119.4	n/a	229.6
$k_5 = k_{flip}$	3.6	3.6	7.4	7.6	52.6	52.4
$k_6 = k_{unflip}$	0.002	0.0020	0.005	0.005	10.3	10.1
$k_7 = k_{artifact}$	0.74 – 2.98	0.44	1.03 – 2.75	0.77	0.01 – 0.20	0.12

Figure S4. Representative global fits to stopped-flow experiments with excess DNA and (A) WT (B) Y162F or (C) Y162A. Lines show the best fits obtained with Berkeley Madonna (see S6), and the plot below shows the residual between the fit and the experimental data. (D) Table summarizing the experimental data (from exponential fits in Table 1 in the main text) described in the main text (Table 1) and the BM (Berkeley Madonna) global analysis at different concentrations of DNA (A–C).

S5. Example of Berkeley Madonna script for global fits to stopped-flow experiments with excess protein

```
METHOD RK4
STARTTIME = 0
STOPTIME = 9.96
DT = 5e-5

d/dt (E[1..3]) = -k1*E[i]*S[i] + k2*ESns[i] + k12*(E2Sns[i] + E2S[i] + E2S1[i]) + k14*(E3Sns[i] + E3S[i] + E3S1[i])

d/dt (S[1..3]) = -k1*E[i]*S[i] + k2*ESns[i]
d/dt (ESns[1..3]) = k12*E2Sns[i] - (k2 + k3 + k11*E[i])*ESns[i] + k1*E[i]*S[i] + k4*ES[i]
d/dt (E2Sns[1..3]) = k11*E[i]*ESns[i] + k14*E3Sns[i] + k4*E2S[i] - (k12 + k5 + k13*E[i])*E2Sns[i]
d/dt (E3Sns[1..3]) = k13*E[i]*E2Sns[i] + k4*E3S[i] - (k14 + k6)*E3Sns[i]
d/dt (ES[1..3]) = k8*ES1[i] - (k4 + k11*E[i] + k7)*ES[i] + k3*ESns[i] + k12*E2S[i]
d/dt (E2S[1..3]) = k11*E[i]*ES[i] + k5*E2Sns[i] + k14*E3S[i] + k8*E2S1[i] - (k12 + k4 + k13*E[i] + k7)*E2S[i]
d/dt (E3S[1..3]) = k6*E3Sns[i] + k13*E[i]*E2S[i] + k8*E3S1[i] - (k14 + k4 + k7)*E3S[i]
d/dt (ES1[1..3]) = k12*E2S1[i] + k7*ES[i] - (k8 + k11*E[i] + k9)*ES1[i]
d/dt (E2S1[1..3]) = k11*E[i]*ES1[i] + k7*E2S[i] + k14*E3S1[i] - (k8 + k13*E[i] + k9 + k12)*E2S1[i]
d/dt (E3S1[1..3]) = k13*E[i]*E2S1[i] + k7*E3S[i] - (k8 + k14 + k9)*E3S1[i]
d/dt (ESc[1..3]) = k9*ES1[i] + k9*E2S1[i] + k9*E3S1[i]

init S[1..3] = Sub
Sub = 50e-9
init ESns[1..3] = 0
init E2Sns[1..3] = 0
init E3Sns[1..3] = 0
init ES[1..3] = 0
init E2S[1..3] = 0
init E3S[1..3] = 0
init ES1[1..3] = 0
init E2S1[1..3] = 0
init E3S1[1..3] = 0
init ESc[1..3] = 0

init E[1] = E1
init E[2] = E2
init E[3] = E3
E1 = 150e-9
E2 = 250e-9
E3 = 400e-9

k1 = 1.1e9
k2 = 48
k3 = 85
k4 = 30
k5 = 2*k3
```

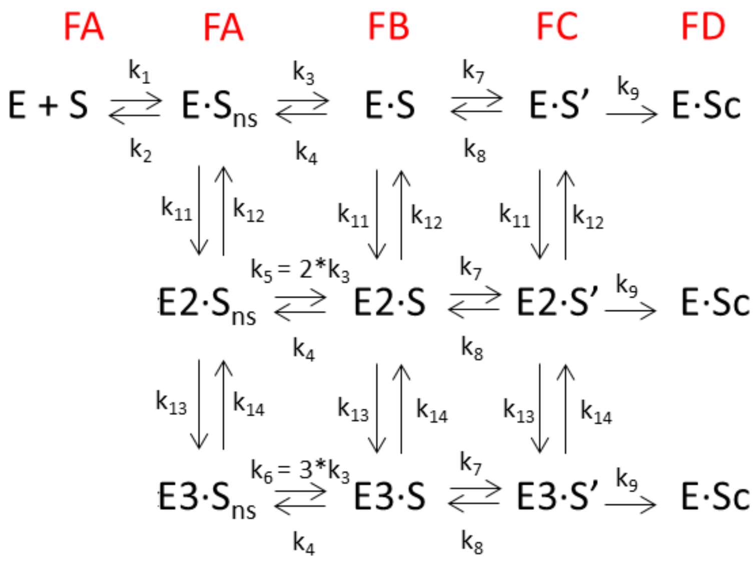

Scheme for multiple proteins binding, where E = AAG, S = DNA substrate, ESns = nonspecific complex, ES = initial recognition complex, ES' = flipped-out specific recognition complex, ESc = artifact phase. E2 assumes binding of two proteins, while E3 is binding of three proteins. To fit this mechanism to fluorescence data, we also define the fluorescence of each species (FA, FB, FC, FD).

k6 = 3*k3
k7 = 3.6
k8 = 0.002
k9 = 0.1
k11 = k1
k12 = k2
k13 = k1
k14 = k2

F[1..3] = OS[i] + FA[i]*(S[i] + ESns[i] + E2Sns[i] + E3Sns[i]) + FB[i]*(ES[i] + E2S[i] + E3S[i]) + FC[i]*(ES1[i] + E2S1[i] + E3S1[i]) + FD[i]*ESc[i]

F1 = F[1]
F2 = F[2]
F3 = F[3]
FA[1] = FA1
FA[2] = FA2
FA[3] = FA3
FB[1] = FB1
FB[2] = FB2
FB[3] = FB3
FC[1] = FC1
FC[2] = FC2
FC[3] = FC3
FD[1] = FD1
FD[2] = FD2
FD[3] = FD3

FA1 = 2.0e8
FA2 = 4.0e8
FA3 =5.0e8
FB1 = 6.0e8
FB2 = 6.3e8
FB3 = 6.4e8
FC1 = 2.0e7
FC2 = 5.0e7
FC3 = 9.0e7
FD1 = 5.0e6
FD2 = 1e7
FD3 = 1.5e7

OS[1] = OS1
OS[2] = OS2
OS[3] = OS3
OS1 = 102.3
OS2 = 101.8
OS3 = 102.5

S6. Example of Berkeley Madonna script for global fits to stopped-flow experiments with excess DNA

```
METHOD RK4
STARTTIME = 0
STOPTIME = 9.96
DT = 5e-5

d/dt (E[1..3]) = -k1*E[i]*S[i] + k2*ESns[i]
d/dt (S[1..3]) = k2*ESns[i] - k1*E[i]*S[i]
d/dt (ESns[1..3]) = k4*ES[i] - (k2 + k3)*ESns[i] + k1*E[i]*S[i]
d/dt (ES1[1..3]) = -k6*ES1[i] + k5*ES[i]  - k7*ES1[i]
d/dt (ES[1..3]) = k6*ES1[i] - (k4 + k5)*ES[i] + k3*ESns[i]
d/dt (ESc[1..3]) = k7*ES1[i]

init E[1..3] = En
En = 100e-9
init ESns[1..3] = 0
init ES[1..3] = 0
init ES1[1..3] = 0
init ESc[1..3] = 0

init S[1] = S1
init S[2] = S2
init S[3] = S3
S1 = 200e-9
S2 = 600e-9
S3 = 900e-9

k1 = 5e9
k2 = 53
k3 = 105
k4 = 18
k5 = 3.6
k6 = 0.002
k7 = 1.1

F[1..3] = OS[i] + FA[i]*S[i] + FA[i]*ESns[i] + FB[i]*ES[i] + FC[i]*ES1[i] + FD[i]*ESc[i]

F1 = F[1]
F2 = F[2]
F3 = F[3]

FA[1] = FA1
FB[1] = FB1
FC[1] = FC1
FD[1] = FD1
FA1 = 3e8
FB1 = 7e8
FC1 = 3e6
FD1 = 1e6
FA[2] = FA2
```

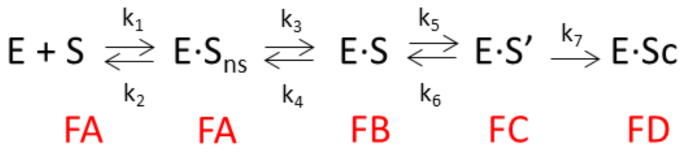

Scheme for binding of a single protein, where E = AAG, S = DNA substrate, ESns = nonspecific complex, ES = initial recognition complex, ES' = flipped-out specific recognition complex, ESc = artifact phase. To fit this mechanism to fluorescence data, we also define the fluorescence of each species (FA, FB, FC, FD).

FB[2] = FB2
FC[2] = FC2
FD[2] = FD2
FA2 = 2e8
FB2 = 3e8
FC2 = 2e6
FD2 = 1e6
FA[3] = FA3
FB[3] = FB3
FC[3] = FC3
FD[3] = FD3
FA3 = 1e8
FB3 = 2e8
FC3 = 2.5e6
FD3 = 1e6

OS[1] = OS1
OS[2] = OS2
OS[3] = OS3
OS1 = 73
OS2 = 30
OS3 = 20

Supplementary References

1. O'Brien PJ & Ellenberger T (2003) Human alkyladenine DNA glycosylase uses acid-base catalysis for selective excision of damaged purines. *Biochemistry* 42(42):12418-12429.
2. Hedglin M & O'Brien PJ (2008) Human alkyladenine DNA glycosylase employs a processive search for DNA damage. *Biochemistry* 47(44):11434-11445.
3. Wolfe AE & O'Brien PJ (2009) Kinetic mechanism for the flipping and excision of 1,N(6)-ethenoadenine by human alkyladenine DNA glycosylase. *Biochemistry* 48(48):11357-11369.
4. Hendershot JM, Wolfe AE, & O'Brien PJ (2011) Substitution of active site tyrosines with tryptophan alters the free energy for nucleotide flipping by human alkyladenine DNA glycosylase. *Biochemistry* 50(11):1864-1874.
5. Kozlov AG & Lohman TM (2002) Stopped-flow studies of the kinetics of single-stranded DNA binding and wrapping around the Escherichia coli SSB tetramer. *Biochemistry* 41(19):6032-6044.
6. Lau AY, Scharer OD, Samson L, Verdine GL, & Ellenberger T (1998) Crystal structure of a human alkylbase-DNA repair enzyme complexed to DNA: mechanisms for nucleotide flipping and base excision. *Cell* 95(2):249-258.
7. Scharer OD, Nash HM, Jiricny J, Laval J, & Verdine GL (1998) Specific binding of a designed pyrrolidine abasic site analog to multiple DNA glycosylases. *J Biol Chem* 273(15):8592-8597.
8. Hsieh J, Walker SC, Fierke CA, & Engelke DR (2009) Pre-tRNA turnover catalyzed by the yeast nuclear RNase P holoenzyme is limited by product release. *RNA* 15(2):224-234.
